# Risk of Depression With Glucagon-Like Peptide-1 (GLP-1) Receptor Agonists Compared to Other Antidiabetic Medications in Adults With Type 2 Diabetes: A Systematic Review and Meta-Analysis

**DOI:** 10.7759/cureus.106972

**Published:** 2026-04-13

**Authors:** Genesis M Ferrer Zavala, Cesar F Caro Rodriguez, Paula Carrion Carrion, Lesly Martinez Artiga, Zuleika Y Morales del Cid, Jia Ee Chia, Carlos Valladares

**Affiliations:** 1 Faculty of Medicine, University of Zulia, Maracaibo, VEN; 2 Faculty of Medicine, Universidad Nuestra Señora de La Paz, La Paz, BOL; 3 Faculty of Medicine, Anáhuac Cancún University, Cancún, MEX; 4 Faculty of Medicine, Universidad Evangelica de El Salvador, San Salvador, SLV; 5 Faculty of Medicine, Latin University of Panama, Panama City, PAN; 6 Internal Medicine, Texas Tech University Health Sciences Center El Paso, El Paso, USA; 7 Pulmonary and Critical Care, University of Miami - Miller School of Medicine, Miami, USA

**Keywords:** anxiety, depression, glucagon-like peptide-1 receptor agonist (glp-1 ra), psychiatry and mental health, types 2 diabetes

## Abstract

Glucagon-like peptide-1 receptor agonists (GLP-1 RAs) are widely used for the management of type 2 diabetes mellitus (T2DM) due to their metabolic and cardiovascular benefits; however, concerns have emerged regarding potential neuropsychiatric effects, including depression. This systematic review and meta-analysis aimed to evaluate the association between GLP-1 RA use and the risk of developing depression in adults with T2DM. A systematic search of PubMed, Embase, Scopus, Web of Science, and the Cochrane Library was conducted from database inception through April 2025 to identify randomized controlled trials and observational studies comparing GLP-1 RAs with other antidiabetic therapies. The primary outcome was incident depression. Secondary outcomes included anxiety-related outcomes and reported adverse events. Risk of bias was assessed using the Risk of Bias in Non-randomized Studies of Interventions (ROBINS-I) tool and the Newcastle-Ottawa Scale, where appropriate. Pooled hazard ratios (HRs) with 95% confidence intervals (CIs) were calculated using a random-effects model. Nine studies involving 1,735,325 patients met the inclusion criteria, of which five contributed to the quantitative synthesis. The meta-analysis demonstrated no statistically significant difference in the risk of incident depression between GLP-1 RA users and those receiving other antidiabetic agents (HR 1.05; 95% CI 0.97-1.14), with substantial heterogeneity observed (I² = 83.2%). Sensitivity analysis showed that exclusion of one influential study increased the pooled estimate to a statistically significant hazard ratio (HR 1.09; 95% CI 1.01-1.19; p = 0.035). Limited evidence suggested potential benefits in anxiety-related outcomes in some studies, although findings were heterogeneous and not amenable to quantitative synthesis. Reported adverse events were predominantly gastrointestinal. Overall, the available evidence suggests that GLP-1 RA use is not associated with an increased risk of depression in adults with type 2 diabetes, though heterogeneity across studies highlights the need for further prospective research with standardized psychiatric outcome assessment.

## Introduction and background

Type 2 diabetes mellitus (T2DM) is a chronic metabolic condition characterized by persistent hyperglycemia resulting from impaired insulin secretion, insulin resistance, or a combination of both. It represents a major and growing global health burden, affecting hundreds of millions of individuals worldwide. Current estimates indicate that more than 1 in 10 adults globally live with diabetes, with projections suggesting a substantial increase in prevalence over the coming decades, largely driven by cases of T2DM [1,2].

In addition to its metabolic complications, T2DM is frequently accompanied by psychiatric comorbidities, most notably depression [3]. The coexistence of diabetes and depression has been attributed to a complex interplay of biological, psychological, and social factors. Proposed mechanisms include chronic low-grade inflammation, dysregulation of the hypothalamic-pituitary-adrenal axis, glycemic variability, and the psychological demands associated with long-term disease management [4,5]. Depression in individuals with T2DM has been associated with poorer self-care behaviors, reduced adherence to treatment, and unfavorable health outcomes [6-8].

According to the Diagnostic and Statistical Manual of Mental Disorders, Fifth Edition (DSM-5), a Major Depressive Episode is diagnosed when an individual experiences at least two weeks of depressed mood or loss of interest or pleasure, accompanied by additional cognitive, emotional, or somatic symptoms that cause clinically significant distress or functional impairment [9]. Given its prevalence and impact, depression represents an important consideration in the comprehensive care of patients with chronic medical conditions such as T2DM [10,11].

Anxiety disorders are among the most prevalent psychiatric conditions worldwide and are characterized by excessive fear, persistent worry, hyperarousal, restlessness, impaired concentration, sleep disturbance, and somatic symptoms such as palpitations or muscle tension [9]. Clinically, anxiety disorders include generalized anxiety disorder, panic disorder, and phobia-related disorders, which are associated with significant functional impairment and reduced quality of life [12]. Anxiety disorders are commonly comorbid with diabetes, and prior research has described a potential bidirectional relationship in which psychological stress and neuroendocrine alterations may influence metabolic outcomes [13,14]. Grigsby et al. found that 40% of patients with diabetes have heightened anxiety symptoms [15].

Glucagon-like peptide-1 receptor agonists (GLP-1 RAs) are a class of antidiabetic medications widely used for the management of T2DM due to their favorable effects on glycemic control, body weight, and cardiovascular outcomes. These agents exert their therapeutic effects by enhancing glucose-dependent insulin secretion, suppressing glucagon release, and slowing gastric emptying. Commonly prescribed GLP-1 RAs include semaglutide, liraglutide, dulaglutide, exenatide, lixisenatide, and albiglutide [16].

Beyond their metabolic actions, GLP-1 receptors are expressed in several regions of the central nervous system, prompting interest in the potential neuropsychiatric effects of GLP-1 RA therapy. Preclinical and clinical studies have explored interactions between GLP-1 signaling and pathways involved in neuroinflammation, stress regulation, and neuroplasticity [17]. At the same time, concerns have been raised regarding possible psychiatric adverse effects, including mood changes and suicidal ideation, leading to uncertainty about the overall mental health impact of these medications [18,19].

Given the widespread and increasing use of GLP-1 receptor agonists and the high prevalence of depression among individuals with T2DM, a clear understanding of the relationship between GLP-1 RA therapy and depression outcomes is clinically important. Therefore, this systematic review and meta-analysis aimed to synthesize available data to evaluate the association between GLP-1 RA use and the risk of developing depression in adults with type 2 diabetes mellitus, compared with other antidiabetic therapies. Secondary objectives included the assessment of anxiety-related outcomes and commonly reported adverse effects associated with GLP-1 RA use.

## Review

Methods

A systematic review was conducted using the Preferred Reporting Items for Systematic Reviews and Meta-Analyses (PRISMA 2020) guidelines [20]. The protocol was registered with the International Prospective Register of Systematic Reviews (PROSPERO; CRD420251059518).

Inclusion and Exclusion Criteria

Studies that consisted of primary clinical data on the use of GLP-1 agonists in the treatment of diabetes mellitus type 2, and the risk of developing depression or initiation of antidepressant therapy in comparison to other anti-diabetic medications were included. Randomized controlled trials (RCTs) and cohort studies were included in the inclusion criteria. The primary outcome was the risk of the development of depression. The secondary outcomes included the presence of anxiety, measured using the Generalized Anxiety Disorder-7 (GAD-7) score or other scales that measure anxiety and side effects among GLP-1 RA users.

Articles were excluded from analysis if they were case reports, case series, systematic reviews, meta-analyses, narrative reviews, editorials, letters, or commentaries. Articles were not excluded based on the nation of origin, but were excluded if they involved specific population characteristics, such as pediatric patients (under 18 years of age), individuals without type 2 diabetes, pregnant patients, or those with a prior diagnosis of depression or prior use of antidepressant therapy. Articles were excluded if they were not in English. Studies were not differentiated based on the dose of GLP-1 agonist used. However, studies were excluded if they lacked a comparator group, did not report on the incidence of depression, changes in depressive symptoms, or antidepressant use, or focused exclusively on anxiety or other psychiatric conditions without depression-related outcomes. Additionally, studies still in progress, without any reported data, or animal, in vitro studies, or non-human research, were also excluded.

Information Sources and Search Strategy

This systematic review utilized five medical databases, including PubMed, Embase, Scopus, Web of Science, and the Cochrane Library. Studies published from database inception through April 4, 2025, were considered; this search was conducted by CV. The search strategy incorporated both controlled vocabulary and free-text terms related to glucagon-like peptide-1 receptor agonists (including specific agents), depression and other mental health outcomes, and type 2 diabetes mellitus. The full electronic search strategy is provided in the Appendices.

Duplicate studies were identified and resolved utilizing Rayyan.ai online software [21]. Following the identification of duplicate articles, one reviewer (GF) manually sorted through the retrieved articles to ensure no further duplicates existed.

Study Selection

Once duplicates were identified and sorted through, title and abstract analysis were conducted to determine inclusion. Following the title and abstract analysis, a full-text appraisal was completed by three trained reviewers (GF, PC, CC). In the event of a debate over the inclusion of a study, a fourth reviewer was brought in to break ties. Studies determined to be eligible for data analysis (both quantitative and qualitative) were subjected to data extraction.

Data Collection and Analysis

Data were independently extracted by multiple reviewers using a standardized data extraction form structured according to the PICO (Participants, Intervention, Comparator, Outcomes) framework. Extracted variables included study design, sample size, demographic characteristics, intervention and comparator therapies, follow-up duration, and reported depression outcomes, including incident depression (new-onset cases), changes in symptom severity, or diagnostic outcomes, as reported by the primary studies. Secondary outcomes included anxiety-related outcomes, defined as clinically relevant anxiety measures such as anxiety diagnoses based on diagnostic codes, scores from validated instruments (e.g., GAD-7, Hospital Anxiety and Depression Scale - Anxiety subscale (HADS-A)), or anxiety-related healthcare utilization, as reported in the primary studies.

Outcome data were extracted as reported in the original studies. This review did not administer, reproduce, or use any clinical questionnaires or instruments. Depression outcomes were identified using diagnostic codes or validated symptom scale scores, as reported by the primary study authors. In some studies, antidepressant initiation was used as a proxy for depression. No proprietary instruments were used directly in this study.

Statistical Analysis

When sufficient data were available, a meta-analysis was conducted to evaluate the association between GLP-1 receptor agonist use and the risk of incident depression. Hazard ratios (HRs) with corresponding 95% confidence intervals (CIs) were extracted or derived from individual studies. Pooled estimates were calculated using a random-effects DerSimonian-Laird model to account for between-study heterogeneity, incorporating both within-study sampling error and between-study variation to provide a more conservative estimate of effect [22]. Statistical significance was defined as a p-value < 0.05, which corresponds to a 95% CI that does not include the null value of 1.00.

Statistical heterogeneity was assessed using the I² statistic, τ², H², and Cochran’s Q test. Sensitivity analyses were performed using a leave-one-out approach to evaluate the influence of individual studies on pooled estimates. Publication bias was explored using visual inspection of funnel plots. All analyses were conducted using R version 4.3.2 (R Foundation for Statistical Computing, Vienna, Austria) [23].

Quality of Evidence Rating and Risk of Bias Assessment

Risk of bias was independently assessed by three reviewers using design-specific validated tools. Non-randomized interventional studies were evaluated using the Risk of Bias in Non-randomized Studies of Interventions (ROBINS-I) tool [24,25]. Cohort studies were assessed using the Newcastle-Ottawa Scale (NOS) for cohort studies [26]. Analytical cross-sectional studies were evaluated using an adapted Newcastle-Ottawa Scale for cross-sectional studies (NOS-xs) [27].

Graphical summaries of risk of bias judgments were generated using the robvis (Risk of Bias Visualization) tool [28]. Detailed itemized NOS and NOS-xs assessments, including total scores and domain-level ratings, are provided in Appendices 1 and 2. Disagreements were resolved by consensus after discussion among the reviewers.

A formal GRADE (Grading of Recommendations Assessment, Development, and Evaluation) assessment was not performed; however, risk of bias was rigorously evaluated at the individual study level using validated methodological instruments.

Results

A search across five databases yielded a total of 944 articles. After removing 426 duplicates using Rayyan.ai’s automatic deduplication tool, 697 unique records remained. These were screened by title and abstract, resulting in 45 articles selected for full-text review. Of these, 36 were excluded for the following reasons: 10 due to wrong study design, such as narrative reviews, editorials, or studies that were neither RCTs nor observational studies (e.g., retrospective cohorts, case-control studies, or case series); 8 were excluded due to wrong publication type, consisting of conference abstracts, protocols, or erratum notices without full data; 11 due to irrelevant outcomes, such as failure to assess depression, its risk factors, or use of unclear or unvalidated scales; 3 used the wrong intervention drug (not a GLP-1 receptor agonist); 1 employed an inappropriate comparator (not another antidiabetic agent); 2 being published in non-English languages (Chinese) and 1 was excluded because of wrong population (teenagers).

**Figure 1:**
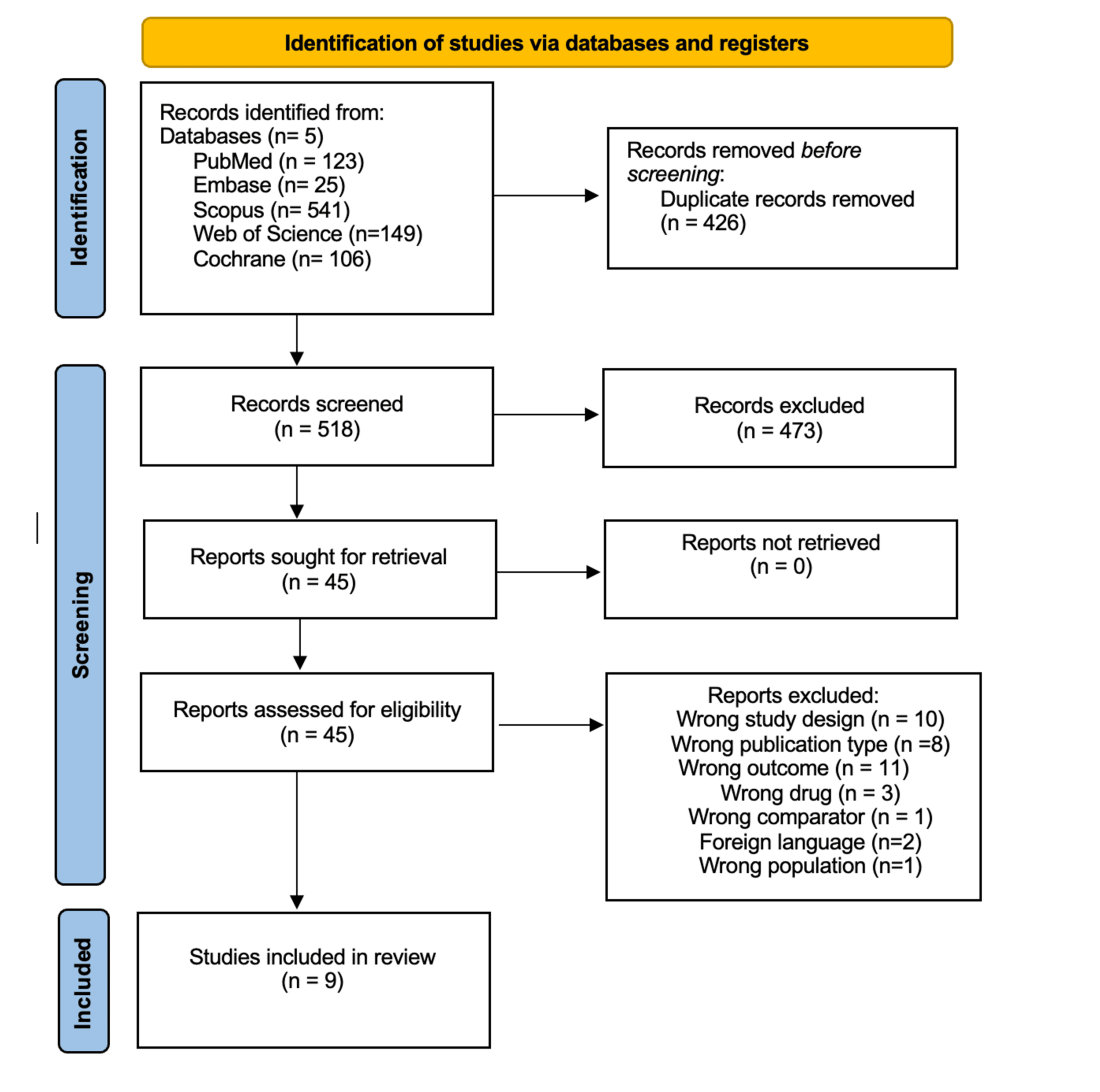
Preferred Reporting Items for Systematic Reviews and Meta-Analyses (PRISMA) flow diagram summarizing the identification, screening, full-text eligibility assessment, and final inclusion of studies in the systematic review and meta-analysis

Ultimately, nine studies met the inclusion criteria and were included in the final analysis. A detailed summary of this process is provided in the PRISMA flow chart (Figure 1).

Study characteristics

Across all included studies, a total of 1,735,325 unique participants with T2DM were analyzed. Among studies reporting treatment-specific cohorts, a total of 1,844,568 GLP-1 RA exposures and 687,348 comparator drug exposures were recorded. Specifically, exposures included 657,992 GLP-1 RA users, 92,218 metformin users, 23,752 insulin users, 58,450 sulfonylurea users, 482,183 dipeptidyl peptidase-4 inhibitor users, 56,341 sodium-glucose cotransporter-2 inhibitor users, and 15,611 thiazolidinedione users.

The number of treatment exposures exceeds the number of unique participants because several studies evaluated multiple treatment groups within the same cohort or allowed medication switching during follow-up; therefore, these values represent treatment exposures rather than individual patients.

Sample sizes varied substantially across studies, ranging from small prospective cohorts to large population-based datasets, and follow-up durations ranged from approximately 3 months to 10 years.

Across the included studies, participants were primarily middle-aged or older adults, with most cohorts reporting a higher proportion of male patients. The GLP-1 receptor agonists examined included semaglutide, liraglutide, dulaglutide, and exenatide. Comparator groups received other standard antidiabetic therapies, including metformin, sulfonylureas, dipeptidyl peptidase-4 inhibitors, sodium-glucose cotransporter-2 inhibitors, thiazolidinediones, and insulin. A comprehensive overview of study characteristics is provided in Table 1.

**Table 1:** Characteristics of studies included in the systematic review The table provides a summary of study design, population characteristics, sample size, duration of follow-up, GLP-1 receptor agonists evaluated, comparator therapies, and reported outcomes across the included studies. T2DM, type 2 diabetes mellitus; CPRD, Clinical Practice Research Datalink; VA, Veterans Affairs; NHIRD, National Health Insurance Research Database; GLP-1 RA, glucagon-like peptide-1 receptor agonist; DPP4i, dipeptidyl peptidase-4 inhibitor; SGLT2i, sodium–glucose cotransporter-2 inhibitor; SU, sulfonylurea; TZD, thiazolidinedione; EHR, electronic health records

Author(s), year	Study Type	Population	Sample Size	Study Duration	Intervention and Methodology	Outcomes and Statistical Findings	Psychometric Tools
Grant et al., 2011 [24]	Prospective cohort (UK, single-center)	Adults with T2DM inadequately controlled on oral therapy	138; GLP-1 RA: female 30, male 41	18 months	Exenatide initiated and followed longitudinally; compared with insulin initiation cohort	GLP-1 therapy associated with improved psychological well-being, treatment satisfaction, and quality of life compared with insulin	Hospital Anxiety and Depression Scale (HADS); Short Form-36 (SF-36); Well-Being Questionnaire-12 (WBQ-12); Diabetes Treatment Satisfaction Questionnaire (DTSQ)
Gamble et al., 2018 [25]	Retrospective cohort (UK CPRD)	Adults with T2DM initiating antidiabetic therapy	136,977; GLP-1 RA: 488	9 years	GLP-1 RA initiators identified from primary care records; matched using propensity scores with cohorts initiating sulfonylureas, thiazolidinediones, or insulin	Depression/self-harm risk: HR 1.25 (95% CI 0.63–2.50) vs. SU; HR 1.18 (95% CI 0.53–2.65) vs. TZD; HR 1.07 (95% CI 0.39–2.94) vs. insulin	Clinical diagnostic codes
De Giorgi et al., 2024 [29]	Retrospective cohort (TriNetX, USA)	Adults with T2DM	130,352; GLP-1 RA: 23,386	12 months	Semaglutide initiators identified from EHR; matched 1:1 using propensity scores with DPP4i, SGLT2i, or SU users	Depression risk: HR 0.92 (95% CI 0.84–1.01) vs. DPP4i; HR 0.99 (95% CI 0.90–1.09) vs. SGLT2i; HR 0.97 (95% CI 0.88–1.07) vs. SU	ICD-10 diagnosis codes
Chen et al., 2025 [30]	Retrospective multicenter cohort (TriNetX)	Adults with T2DM on monotherapy	359,787; GLP-1 RA: 41,846	8 years	GLP-1 RA users identified from EHR and matched using propensity scores with metformin, DPP4i, or SGLT2i users	Depression risk: HR 1.06 (95% CI 0.89–1.26) vs. metformin; HR 0.68 (95% CI 0.58–0.80) vs. DPP4i; HR 1.00 (95% CI 0.78–1.27) vs. SGLT2i	ICD-10 diagnosis codes
Tagliapietra et al., 2024 [31]	Retrospective cohort (VA database)	Adults with T2DM	139,608; female 101,866; male 32,619; GLP-1 RA: 34,130; comparator: 105,478	7 years	GLP-1 RA identified from pharmacy records; compared with DPP4i or SGLT2i users using propensity weighting	Depression risk: HR 0.98 (95% CI 0.95–1.01)	Diagnostic codes
Tsai et al., 2022 [32]	Retrospective cohort (Taiwan NHIRD)	Adults with T2DM	53,456; female 23,915; male 18,851; GLP-1 RA: 10,690; comparator: 39,194	7 years	GLP-1 RA exposure defined as >180 days; compared with non-GLP-1 users using propensity matching	Depression: HR 0.85 (95% CI 0.66–1.11); anxiety: HR 0.70 (95% CI 0.57–0.85)	Claims-based diagnostic codes
Wium-Andersen et al., 2022 [33]	Nationwide cohort + nested case-control	Adults with and without T2DM	139,996; female 8,549; male 73,869	10 years	GLP-1 RA exposure identified via prescription registry (time-varying); compared with other antidiabetic drug users	Depression risk: HR 0.77–0.88 (95% CI 0.71–0.97)	Registry diagnosis and antidepressant prescription
Eren-Yazicioglu, 2021 [34]	Prospective cohort (Turkey)	Obese adults with T2DM	43; female 23; male 18	3 months	Exenatide administered subcutaneously; compared with the non-exenatide group	Significant reduction in depression and affective scores	Validated depression and affective scales
Do et al., 2025 [35]	Retrospective cohort (US claims database)	Adults with T2DM initiating therapy	774,968; GLP-1: female 209,991/male 245,875; comparator: female 156,058/male 163,044	12 months	GLP-1 RA users identified in the claims database; matched using propensity scores with DPP4i users	Lower healthcare utilization related to depression and anxiety in the GLP-1 group	Administrative diagnostic codes

Full-Text Articles Excluded From Analysis

Of the articles subjected to full-text appraisal, 426 were excluded from the final analysis. In some cases, studies were excluded due to insufficient outcome data on depression, lack of a comparator group, or absence of GLP-1 receptor agonist-specific results. However, given the limited number of high-quality studies exploring the neuropsychiatric impact of GLP-1 RAs in adults with type 2 diabetes, we considered retaining studies with less conventional designs (e.g., nested case-control) if they provided relevant data on depression outcomes, antidepressant use, or psychiatric safety. Inclusion was determined based on their potential to contribute meaningful clinical insights despite methodological limitations.

Results of quality of evidence rating and risk of bias assessment

**Figure 2:**
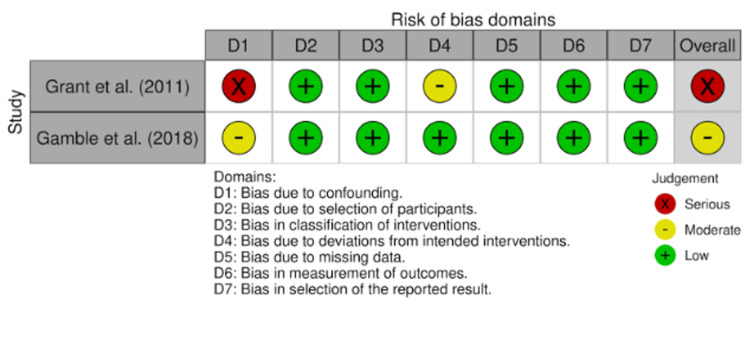
ROBINS-I traffic light plot displaying domain-level risk of bias judgments across included retrospective studies ROBINS-I, Risk of Bias in Non-randomized Studies of Interventions Studies [24-25]

**Figure 3:**
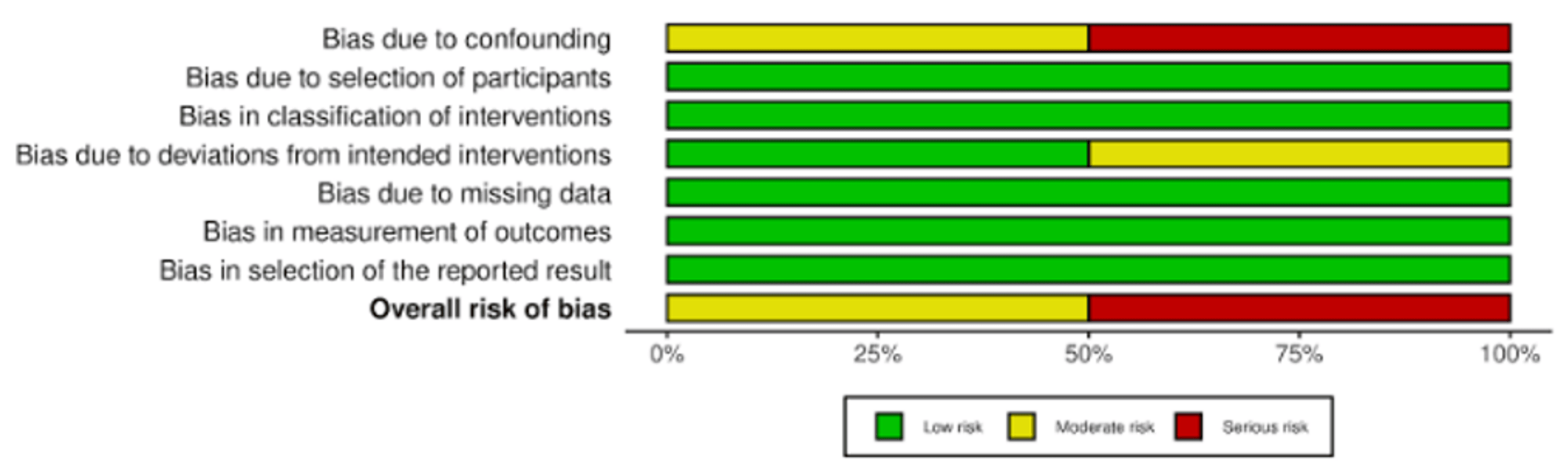
Summary plot of overall risk of bias using ROBINS-I across all retrospective studies ROBINS-I, Risk of Bias in Non-randomized Studies of Interventions

Risk of bias was evaluated according to the study design. Traditional cohort studies were assessed using the NOS, while studies evaluating comparative treatment effects were assessed using the ROBINS-I tool. Graphical summaries are presented in Figures 2-3, and detailed scoring is provided in Appendices 1 and 2.

The NOS assessment showed variability in methodological quality across studies. Scores in the selection domain ranged from moderate to high quality, while the comparability domain indicated adequate adjustment for key confounders in most studies. Outcome and exposure assessments were generally rated as high quality, and follow-up was adequate in most studies. Overall, most studies were classified as having moderate to high methodological quality according to the Newcastle-Ottawa Scale.

The ROBINS-1 assessment showed heterogeneity in risk of bias domains. Confounding bias was considered serious in some studies, whereas selection bias and classification of interventions were consistently rated as low risk. Outcome measurement bias was also low across studies. Bias due to deviations from intended interventions and missing data was rated as moderate in certain studies, while selective reporting bias remained low. Overall, one study was judged to have a serious overall risk of bias, and another a moderate overall risk of bias.

Overview of outcomes

A total of nine studies were included, comprising retrospective cohorts, prospective observational studies, and interventional trials [24,25,29-35]. Across the included studies, the mean age of participants was approximately 65 years, with most cohorts consisting predominantly of male participants. Follow-up durations ranged from 3 months to 10 years, reflecting variability in study design and exposure windows.

Depression

Five studies provided quantitative data for pooled analysis [25,29-32]. The pooled HR for incident depression in GLP-1 RA users compared with other antidiabetic agents was 1.05 (95% CI: 0.97-1.14), indicating no significant difference in depression risk. However, substantial heterogeneity was observed (I² = 83.2%). Sensitivity analyses demonstrated that exclusion of one of the studies shifted the pooled HR to 1.09 (95% CI: 1.01-1.19) [29].

Anxiety and psychiatric well-being

Three studies assessed anxiety outcomes. Tsai et al. reported a lower incidence of anxiety among GLP-1 RA users compared with non-users [32]. Do et al. described improvements in psychological well-being and reduced emotional distress, though without formal anxiety scale reporting [35]. In contrast, Chen et al. and Wium-Andersen et al. did not evaluate anxiety as an independent endpoint [30,33].

Adverse events

Six studies provided information on adverse effects. De Giorgi et al. [29], Tagliapietra et al. [31], and Do et al. [35] documented nausea and vomiting as the most common complaints, while Eren-Yazicioglu et al. additionally noted reduced appetite and weight loss [34]. Grant et al. and Gamble et al. reported no major psychiatric adverse events or did not emphasize side effects [24,25]. Across studies, adverse events were generally mild to moderate and did not lead to treatment discontinuation in most cases. A summary of findings and population characteristics can be seen in Table 2.

**Table 2:** Summary of findings and population characteristics across included studies GLP-1 RA, glucagon-like peptide-1 receptor agonist; DPP4i, dipeptidyl peptidase-4 inhibitor; SGLT2i, sodium–glucose cotransporter-2 inhibitor; HR, hazard ratio; CI, confidence interval; GI, gastrointestinal; I², inconsistency index Treatment counts represent therapy exposures reported across included studies and may exceed the number of unique participants.

Category	Value
Total number of studies	9
Total number of patients	1,735,325
Mean age (range)	65 years (63-66)
Sex distribution	Male: 34,581 (75.6%); female: 11,178 (24.4%)
Duration of GLP-1 RA use	10–12 months
Number of participants using metformin	92,218
Number of participants using insulin	23,752
Number of participants using sulfonylureas	58,450
Number of participants using DPP4i	482,183
Number of participants using GLP-1 RAs	657,992
Number of participants using SGLT2i	56,341
Number of participants using thiazolidinediones	15,611
Most common side effects	Nausea, vomiting, diarrhea, reduced appetite
Depression outcome	Incident depression – pooled HR = 1.05 (95% CI: 0.97–1.14); high heterogeneity (I² = 83.2%)
Anxiety outcomes	Lower anxiety incidence in GLP-1 RA users; reduced healthcare utilization
Adverse events	GI side effects most common; generally mild to moderate; rare discontinuation

Meta-analysis of the depression outcomes

**Figure 4:**
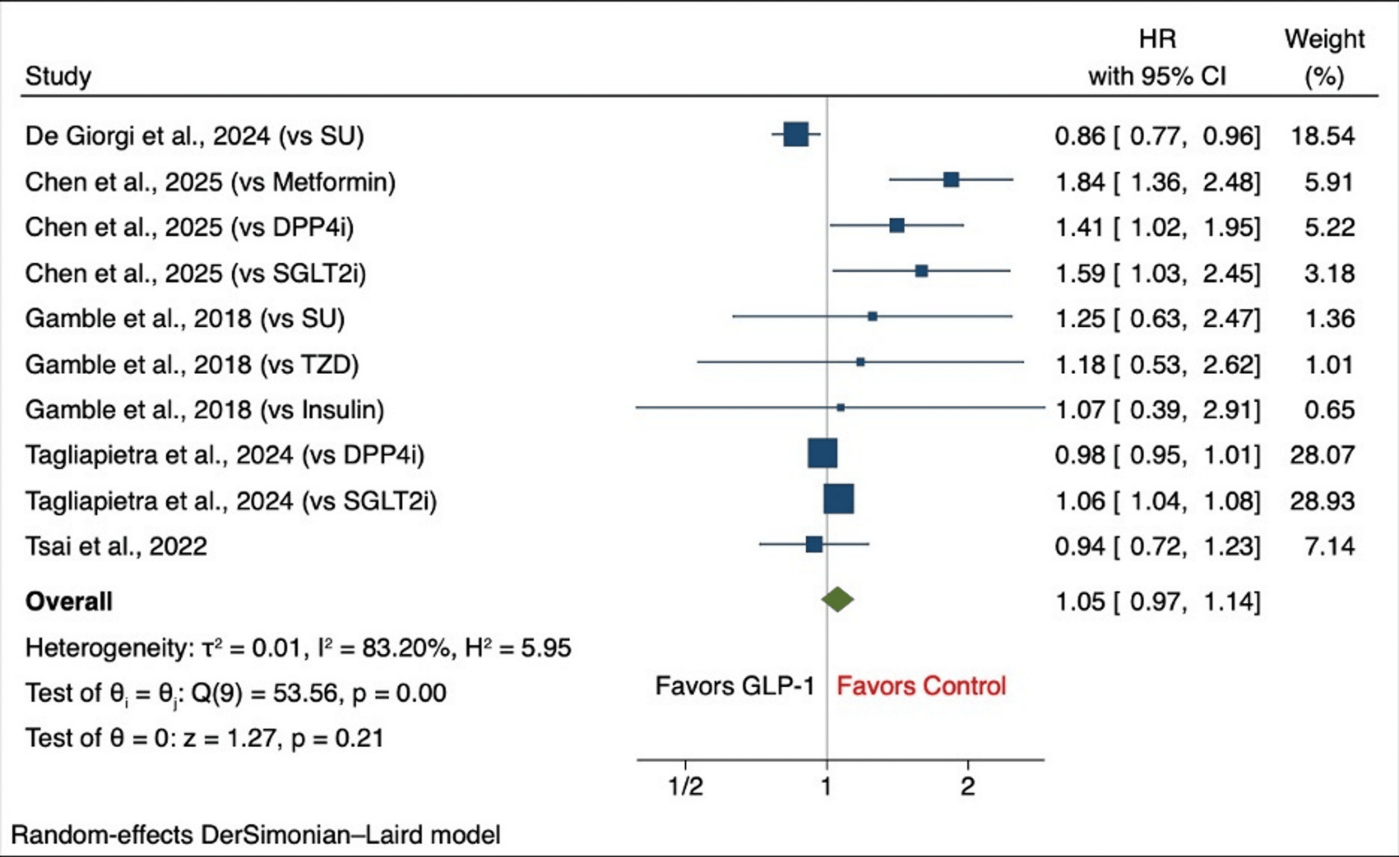
Forest plot of the association between glucagon-like peptide-1 receptor agonist (GLP-1 RA) use and incident depression The figure displays hazard ratios (HRs) with 95% confidence intervals (CIs) comparing GLP-1 RAs with other antidiabetic agents across included studies [25,29-32]. A random-effects meta-analysis was performed using the DerSimonian–Laird method. HR, hazard ratio; CI, confidence interval; SU, sulfonylurea; TZD, thiazolidinedione; DPP4i, dipeptidyl peptidase-4 inhibitor; SGLT2i, sodium–glucose cotransporter-2 inhibitor

Five studies contributed quantitative data to the meta-analysis evaluating the association between glucagon-like peptide-1 receptor agonist use and incident depression. The pooled analysis included a total of 820,180 participants. Using a random-effects model, the pooled hazard ratio for incident depression among individuals treated with GLP-1 receptor agonists compared with other antidiabetic therapies was 1.05 (95% CI: 0.97-1.14), indicating no statistically significant difference between groups. Individual and pooled effect estimates are presented in Figure 4.

Heterogeneity

Considerable heterogeneity was identified among the included studies. The I² value of 83.2% indicated that the majority of the variability in effect estimates was due to between-study differences rather than random error. Between-study variance was estimated at τ² = 0.01, and the H² statistic was 5.95, further demonstrating substantial inconsistency across results. Cochran’s Q test confirmed significant heterogeneity (Q = 53.56, df = 9, p < 0.001). This variability may be explained by differences in study design, participant characteristics, follow-up duration, and the comparator antidiabetic therapies used.

Sensitivity Analysis

**Figure 5:**
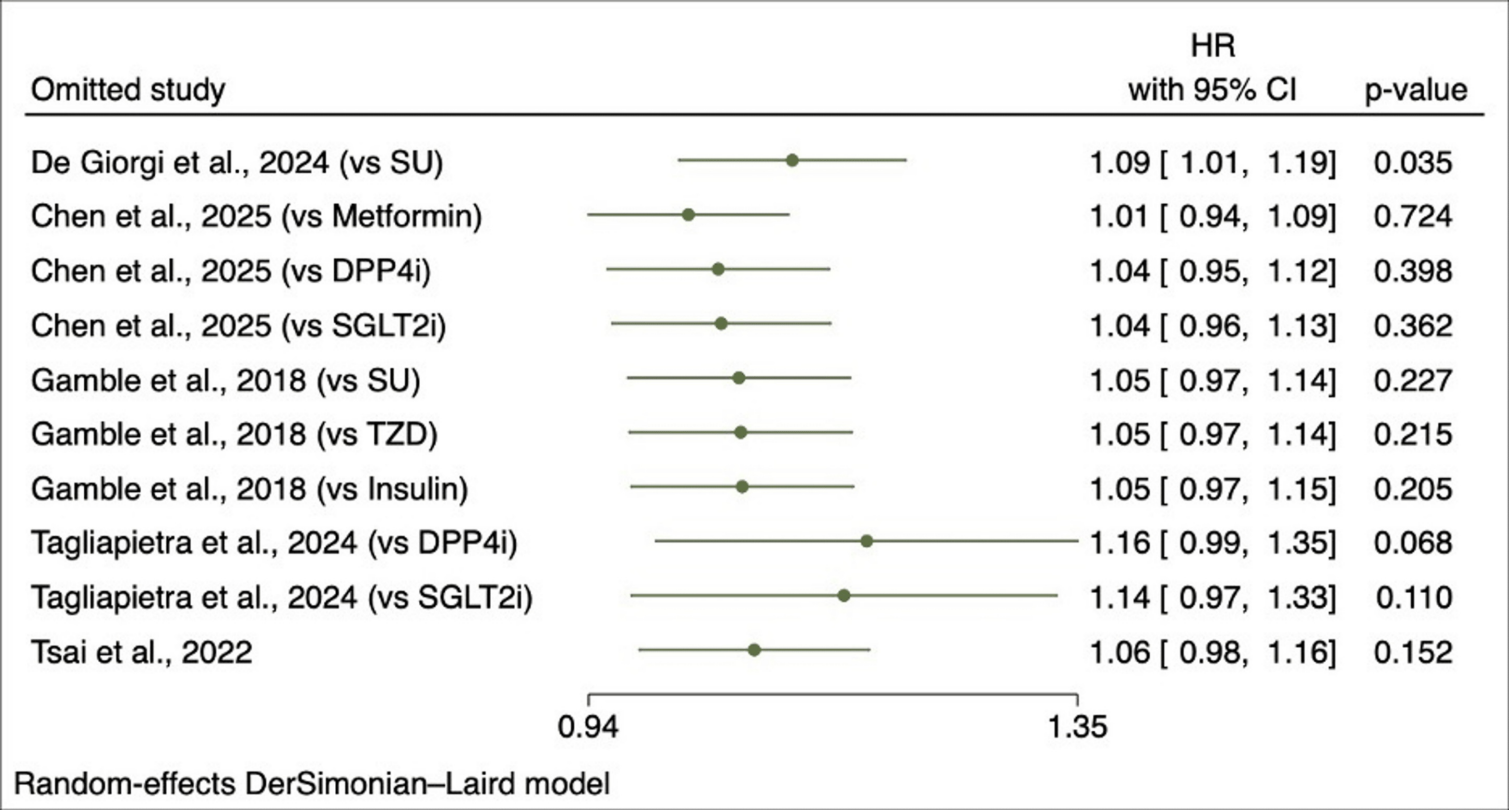
Sensitivity analysis using leave-one-out method Each point represents the pooled hazard ratio (HR) and 95% confidence interval (CI) obtained after sequential exclusion of individual studies from the meta-analysis [25,29-32]. SU, sulfonylurea; TZD, thiazolidinedione; DPP4i, dipeptidyl peptidase-4 inhibitor; SGLT2i, sodium–glucose cotransporter-2 inhibitor

A leave-one-out sensitivity analysis was performed to evaluate the stability of the pooled effect estimate (Figure 5). Removal of one influential study increased the pooled hazard ratio to 1.09 (95% CI: 1.01-1.19; p = 0.035). Excluding the remaining studies did not meaningfully change the overall results, with pooled hazard ratios ranging from 1.01 to 1.16 and p-values remaining non-significant (>0.05). Overall, the findings suggest that the meta-analysis results were largely robust but affected by individual study contributions.

Publication Bias

**Figure 6:**
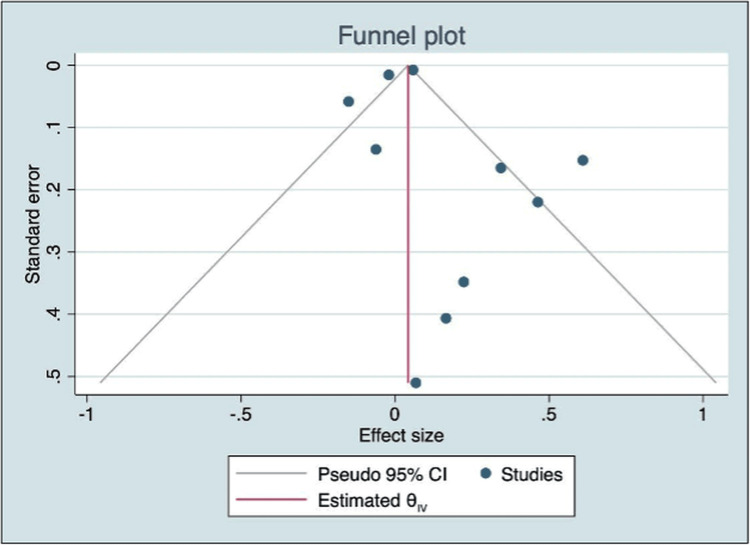
Funnel plot used to examine potential publication bias across included studies The plot displays the distribution of individual study effect estimates against their standard errors, with asymmetry suggesting possible publication bias or small-study effects.

Examination of the funnel plot (Figure 6) showed asymmetry, with a greater concentration of studies on one side of the pooled effect estimate and fewer studies on the other. This distribution may suggest the presence of publication bias or small-study effects, in which smaller studies with non-significant results are less likely to be represented.

Discussion

This systematic review and meta-analysis evaluated the risk of developing depression in patients with type 2 diabetes treated with glucagon-like peptide-1 receptor agonists compared with those receiving other antidiabetic agents. The qualitative synthesis included nine studies comprising a total of 1,735,325 participants. Of these, five studies provided sufficient data for inclusion in the quantitative meta-analysis. Across these five studies, 657,992 participants were exposed to GLP-1 RAs and 728,555 to comparator therapies. The review was conducted in accordance with the PRISMA guidelines.

Findings across studies were inconsistent, with some cohorts indicating a higher risk of depression among GLP-1 RA users and others reporting reduced risk [36]. In contrast, the pooled meta-analysis showed no statistically significant difference in depression risk between individuals treated with GLP-1 RAs and those receiving other antidiabetic therapies (HR: 1.05; 95% CI: 0.97-1.14). The differences between individual cohort results and the pooled estimate may be explained by the larger combined sample size and the limited number of studies reporting statistically significant associations.

Substantial heterogeneity was observed across the included studies, reflecting variability in study design, populations, exposure duration, outcome definitions, and comparator therapies. This heterogeneity is consistent with the inclusion of real-world observational studies, which differ in baseline psychiatric risk, methods of depression ascertainment, and adjustment for confounding factors. Sensitivity analyses demonstrated that the pooled estimate was influenced by a single influential study. Exclusion of the study by De Giorgi et al. shifted the pooled hazard ratio to 1.09 (95% CI: 1.01-1.19), indicating that this study contributed substantially to the observed heterogeneity. Exclusion of other studies did not materially alter the pooled results, suggesting that the overall findings were generally stable but sensitive to individual study contributions [16].

The methodological quality assessment showed overall moderate to high quality across the included studies. Cohort studies evaluated using the Newcastle-Ottawa Scale generally demonstrated adequate selection methods, comparability adjustment, and outcome ascertainment, although some studies reported incomplete follow-up. Studies assessed using the ROBINS-I tool showed low risk in selection and outcome measurement domains, while confounding represented the primary source of bias, with some studies rated as moderate to serious risk.

Our findings are consistent with previous systematic reviews and meta-analyses examining the association between GLP-1 RAs and mental health outcomes. Notably, Silverii et al. (2024) conducted a comprehensive meta-analysis of 31 randomized controlled trials, including 84,713 patients with type 2 diabetes or obesity, to evaluate psychiatric adverse events such as depression, anxiety, and suicidal behavior [37]. In line with our results, they reported no significant increase in psychiatric adverse events among patients treated with GLP-1 RAs compared with controls and found no statistically significant associations with depression, anxiety, or suicidal behavior, with low heterogeneity across trials. These findings support the absence of a strong association between GLP-1 RA therapy and adverse psychiatric outcomes and are broadly consistent with our pooled estimates derived from real-world data [25].

It is important to note that the analysis by Silverii et al. was limited to RCTs, which frequently exclude individuals with pre-existing psychiatric disorders and often involve shorter follow-up periods, potentially limiting generalizability. In contrast, our review incorporated retrospective and observational studies conducted in real-world settings, thereby capturing more diverse patient populations and longer exposure durations. Furthermore, we extended our evaluation beyond depression to include secondary outcomes such as anxiety and reported adverse effects, which are often underreported in RCTs [25].

With respect to anxiety and psychiatric well-being, a limited number of studies assessed these outcomes. Some studies suggested lower rates of anxiety-related outcomes or improvements in psychological well-being among GLP-1 RA users, whereas other studies did not evaluate anxiety as a distinct endpoint [38,39]. Due to heterogeneity in outcome definitions and measurement tools, these findings could not be quantitatively synthesized and should be interpreted cautiously [17,21].

Adverse event reporting across studies was consistent with the established safety profile of GLP-1 RAs. Gastrointestinal symptoms, including nausea, vomiting, diarrhea, reduced appetite, and weight loss, were the most reported adverse effects. Several studies documented nausea and vomiting as predominant side effects, while others additionally reported reduced appetite and weight loss. This finding is consistent with a recent large-scale meta-analysis showing that although gastrointestinal adverse effects are common, they are typically transient and are not associated with long-term neuropsychiatric complications [38,40].

Psychiatric adverse events were rarely reported, and most adverse effects were mild to moderate in severity, with few cases requiring discontinuation of treatment [16,19,22]. Adding to this body of evidence, Cooper et al. (2023) conducted a systematic review and meta-analysis reporting a reduced risk of incident depression associated with GLP-1 RA use in adults with type 2 diabetes. This real-world evidence aligns with the protective trends observed in some of the individual studies included in our review, despite our pooled estimate not reaching statistical significance [41]. Our results are further corroborated by clinical data and regulatory reviews from the FDA and EMA, which found no definitive causal link between GLP-1 receptor agonists and increased suicidal ideation or severe mood disorders [42,43].

Collectively, the available evidence suggests that GLP-1 RAs are not associated with an increased risk of depression and may be safely prescribed from a mental health standpoint in adults with type 2 diabetes. Nevertheless, given the observed heterogeneity and limited data on anxiety and other psychiatric outcomes, continued pharmacovigilance and future high-quality prospective studies are warranted, particularly in populations at higher baseline psychiatric risk.

Limitations

While the findings are informative, several limitations should be considered. First, substantial heterogeneity was present across included studies in terms of patient populations, treatment duration, comparator therapies, and outcome definitions, which may limit the generalizability of the findings. Second, there was significant variability in the assessment of depression outcomes. While the primary meta-analysis was restricted to studies reporting incident depression using diagnostic codes and hazard ratios, other included studies assessed depressive symptoms using validated instruments such as the Patient Health Questionnaire-9 and Hamilton Depression Rating Scale. These instruments differ in structure, scoring systems, and clinical thresholds, limiting their comparability. As a result, studies using symptom scales were not included in the quantitative synthesis. This heterogeneity in outcome measurement represents an important source of variability and may impact the validity and interpretation of the pooled estimates. Third, some included studies used antidepressant initiation as a proxy for depression. This approach may introduce misclassification bias, as these medications are prescribed for multiple indications beyond depression, including anxiety disorders, chronic pain, and sleep disturbances, and therefore may not accurately reflect a confirmed diagnosis of depression. Fourth, most included studies were observational cohort designs and therefore susceptible to residual confounding. Methodological quality varied across studies, as reflected by assessments using the Newcastle-Ottawa Scale for cohort studies and the ROBINS-I tool for non-randomized interventional studies, where confounding represented a key source of potential bias. Additionally, few studies evaluated secondary outcomes such as anxiety, and psychiatric adverse effects were often not described in detail. Finally, follow-up duration was relatively short in several studies, with many assessing outcomes over approximately 10 to 12 months, which may be insufficient to evaluate long-term mental health effects.

Clinical and research implications

The results of this systematic review and meta-analysis suggest that the use of GLP-1 receptor agonists in adults with type 2 diabetes is not significantly associated with an increased or decreased risk of developing depression when compared to other antidiabetic drugs. While some individual studies reported either protective or adverse associations, the pooled data revealed no statistically significant effect. Clinicians should therefore continue prescribing GLP-1 receptor agonists based on their established metabolic and cardiovascular benefits, while remaining attentive to potential neuropsychiatric effects in specific subgroups. Routine screening for depressive symptoms may be particularly prudent following treatment initiation, highlighting the importance of considering a patient’s mental health history when choosing antidiabetic treatments, enabling more individualized care, particularly for those with a background of psychiatric conditions.

From a research perspective, these findings highlight the need for well-designed, prospective investigations to evaluate the neuropsychiatric effects of GLP-1 receptor agonists. High heterogeneity among studies due to variability in depression assessment methods, comparator agents, and population characteristics limits the generalizability of current evidence. Future studies should incorporate standardized diagnostic tools, enroll more diverse patient populations, and assess mental health outcomes as primary endpoints. In addition, exploring the underlying biological pathways, particularly the influence of GLP-1 on neuroinflammation and brain plasticity, may provide critical insights into possible psychiatric effects. Enhanced collaboration between endocrinologists, mental health specialists, and neuroscientists will also be essential to develop more holistic and targeted research approaches in this area.

## Conclusions

This systematic review and meta-analysis indicated that the use of GLP-1 receptor agonists in adults with type 2 diabetes is not significantly associated with either an increased or decreased risk of depression in adults with T2DM, suggesting that GLP-1 RAs are generally safe from a psychiatric standpoint. Clinicians should continue prescribing GLP-1 RAs for their metabolic benefits while monitoring for mental health symptoms in at-risk individuals. Future research should focus on investigating potential long-term neuropsychiatric effects.

## Appendices

Appendix 1

**Table 3:** Newcastle–Ottawa Scale for cross-sectional studies (NOS-xs) PHQ-9, Patient Health Questionnaire-9; PSS, Perceived Stress Scale Total score: 5/9; overall risk of bias: moderate Eren-Yazicioglu et al., 2021 [34]

Domain	Item	Response Category	Stars Awarded
Study sample selection			
1. Representativeness of sample	Selected clinical group (convenience sample from endocrinology clinics)	0	Single-center outpatient recruitment; no random sampling; limited external validity
2. Sample size	Not justified or underpowered	0	A priori estimate required n=45; achieved n=43; no power recalculation reported
Assessment of exposure and outcome			
3. Assessment of exposure (exenatide use)	Gold-standard (medical records + clinical confirmation)	2	Exposure verified via endocrinology clinic records and prescription history
4. Assessment of outcome (depressive symptoms – PHQ-9)	Acceptable validated tool	1	PHQ-9 validated Turkish version; self-report scale, not diagnostic interview
Confounding factors			
5. Adjustment for confounders	Adjusted for some but not all key confounders	1	Adjusted for BMI and PSS; did not adjust for diabetes duration, antidepressant use, metabolic markers
6. Assessment of confounders	Acceptable measurement methods	1	BMI and stress scales validated; confounders measured appropriately

Appendix 2

**Table 4:** Newcastle–Ottawa Scale (cohort studies) S = Selection domain; C = Comparability domain; O = Outcome domain. Each item was scored as 1 (criterion met) or 0 (criterion not met), according to the Newcastle–Ottawa Scale for cohort studies. The maximum possible score was 9 points. Major confounders were predefined a priori as age, sex, baseline psychiatric history, diabetes severity or duration, and relevant metabolic comorbidities; 1 point was awarded if studies adjusted for major confounders (C1), and an additional point was awarded for adjustment of other clinically relevant covariates (C2). Follow-up duration (O2) was considered adequate if sufficient time was allowed for the development of psychiatric outcomes based on study objectives. Adequacy of follow-up (O3) required clear reporting of attrition and minimal loss to follow-up. Total scores were interpreted as follows: 7–9 points, low risk of bias; 4–6 points, moderate risk of bias; 0–3 points, high risk of bias.

Study	S1 Representativeness	S2 Non-Exposed Selection	S3 Exposure Ascertainment	S4 Outcome Absent at Start	C1 Adjusts Major Confounders	C2 Additional Adjustments	O1 Outcome Assessment	O2 Follow-Up Duration	O3 Adequacy of Follow-Up	Total
De Giorgi et al., 2024 [29]	1	1	1	1	1	1	1	1	1	9
Gamble et al., 2018 [25]	1	1	1	1	1	1	1	1	1	9
Do et al., 2025 [35]	1	1	1	1	1	1	1	1	0	8
Tsai et al., 2022 [32]	1	1	1	1	1	1	1	1	1	9
Grant et al., 2011 [24]	0	1	1	1	1	0	1	0	1	6
Wium-Andersen et al., 2022 [33]	1	1	1	1	1	1	1	1	1	9

Appendix 3

**Table 5:** Embase searches

Embase	
Search	Results
("Glucagon-Like Peptide 1 Receptor Agonists" OR "GLP-1 receptor agonist*" OR "glucagon-like peptide-1" OR semaglutide OR liraglutide OR dulaglutide OR exenatide OR lixisenatide OR albiglutide) AND ("depression" OR "depressive disorder" OR "mood disorder" OR "depressive symptoms" OR suicidality OR "suicidal ideation" OR "mental health") AND ("diabetes mellitus, type 2" OR "type 2 diabetes" OR T2DM).	25

Appendix 4

**Table 6:** Scopus searches

Scopus	
Search	Results
("Glucagon-Like Peptide 1 Receptor Agonists" OR "GLP-1 receptor agonist*" OR "glucagon-like peptide-1" OR semaglutide OR liraglutide OR dulaglutide OR exenatide OR lixisenatide OR albiglutide) AND ("depression" OR "depressive disorder" OR "mood disorder" OR "depressive symptoms" OR suicidality OR "suicidal ideation" OR "mental health") AND ("diabetes mellitus, type 2" OR "type 2 diabetes" OR T2DM).	541

Appendix 5 

**Table 7:** Cochrane searches

Cochrane	
Search	Results
("Glucagon-Like Peptide 1 Receptor Agonists" OR "GLP-1 receptor agonist*" OR "glucagon-like peptide-1" OR semaglutide OR liraglutide OR dulaglutide OR exenatide OR lixisenatide OR albiglutide) AND ("depression" OR "depressive disorder" OR "mood disorder" OR "depressive symptoms" OR suicidality OR "suicidal ideation" OR "mental health") AND ("diabetes mellitus, type 2" OR "type 2 diabetes" OR T2DM).	106

Appendix 6

**Table 8:** Web of Science searches

Web of Science	
Search	Results
("Glucagon-Like Peptide 1 Receptor Agonists" OR "GLP-1 receptor agonist*" OR "glucagon-like peptide-1" OR semaglutide OR liraglutide OR dulaglutide OR exenatide OR lixisenatide OR albiglutide) AND ("depression" OR "depressive disorder" OR "mood disorder" OR "depressive symptoms" OR suicidality OR "suicidal ideation" OR "mental health") AND ("diabetes mellitus, type 2" OR "type 2 diabetes" OR T2DM).	149

Appendix 7

**Table 9:** PubMed searches

PubMed	
Search	Results
("Glucagon-Like Peptide 1 Receptor Agonists" OR "GLP-1 receptor agonist*" OR "glucagon-like peptide-1" OR semaglutide OR liraglutide OR dulaglutide OR exenatide OR lixisenatide OR albiglutide) AND ("depression" OR "depressive disorder" OR "mood disorder" OR "depressive symptoms" OR suicidality OR "suicidal ideation" OR "mental health") AND ("diabetes mellitus, type 2" OR "type 2 diabetes" OR T2DM).	123
